# Supplementary material for: In Vitro and In Silico Mechanistic Insights into miR-21-5p-Mediated Topoisomerase Drug Resistance in Human Colorectal Cancer Cells
Source: Biomolecules. 2019 Sep 9;9(9):467. doi: 10.3390/biom9090467 (PMC6769444; doi:10.3390/biom9090467)
Supplement: Supplementary file 1 [file biomolecules-09-00467-s001.zip › Table S1-Genes-CLUE.docx]

**Table S1. The gene list for Connectivity Map (CMap) analysis.** The most upregulated 193 and downregulated 167 genes were inputted to CLUE database to obtain 150 upregulated and 150 downregulated valid genes for query.

| **Upregulated genes** | **Downregulated genes** |
| --- | --- |
| SYNC, ODAM, VASH2, ENTPD3, TMPRSS4, FUT3, S100P, KCNQ1, CHRNA6, DLL3, KLK10, APOA1, FAM163B, GUCY2GP, GGT6, LECT1, LRTOMT, RC3H1, IPCEF1, CCL20, PRH2, NUPR1, CHODL-AS1, CCR9, TDGF1, CTNNA1, CD28, NPSR1, SLITRK2, NPNT, CCDC8, NRG3, C2orf61, TAOK1, SLPI, KLRK1, TVP23A, KCNQ1, BAZ2A, LOC728637, ZNF781, FPR2, MMP25, TSPYL6, SERPINB4, ATP1A4, ECM2, MMP14, SLC6A19, KCNJ18, NMB, FAM211A, GPR85, ORM1, LPGAT1, HSD17B13, OR1F2P, CRYBB2, LOC101060391, ANGPTL1, TRIM77P, TWIST2, SULT1C2, PROS1, FLG, SLC15A5, QRFPR, LOC284688, TTTY3B, LEPREL4, GADL1, C1orf141, CPT1A, RASGRF1, C5orf20, LILRB2, RINL, KIAA1211L, SPATA6, BEGAIN, OPALIN, GALNTL6, C17orf77, CPM, ADAMTS1, MMP16, DCAF12L2, OR7G1, ANKRD24, DAZ3, C13orf35, SKINTL, RPSAP52, SLFNL1, LY96, ASMT, OSGIN2, MLANA, WDR65, LINC00111, DKFZP686I15217, NXPH2, TAS2R7, PRKCB, C1orf87, TCEB1, LRRC32, SAMD9L, NEUROD4, ARHGEF40, C17orf104, MAP1B, PORCN, FLJ45964, MYBPH, MED13L, TTC16, UCA1, COLEC12, IDO2, PHACTR1, JDP2, CATSPERB, SSX2, CALML6, NEU3, SLC1A4, SPTBN2, OR6V1, CRTAM, ZDBF2, LOC441081, LRRIQ1, SLC16A14, PDE11A, WFDC8, PTPRQ, OVOL1, PRKCQ, SYN1, CPLX4, DIAPH2, TNFRSF18, RUFY2, CDHR2, PLA2G4E, FGF23, FSD1L, CACNA1C, SPDYC, NPAP1, ILDR1, LENG9, LHX6, RNLS, ARL15, IFI27L2, KCND3, GP6, C2orf83, SNX30, SNX18, CD80, WTH3DI, RASIP1, SVOPL, UNC5B, VPS13B, DCLK2, CLCN5, CRYGA, VCY1B, ZSCAN23, GFAP, FGR, TNFAIP6, ZFP69, RPRM, TAS2R13, MBNL3, SHISA9, WIPF1, TREM1, UGT8, TMPRSS11F, OR51B2, BTD, NIPSNAP3B, ZNF568, MSRB3, OR5K3, KCNK10, MLLT11, BBS5, SLC15A5, JPH1, PDE5A, C9orf152, RGSL1, WDR64, ARID5A, BTBD7, SLC26A4, ABHD8, POLM, C16orf97, SLC2A13, ACTN1, BPY2, FAM90A1, TTC28, CCL17, ZNF287, OXGR1, SLC16A1, APBA2, LOC730268, BACH2, HPYR1, ANKRD18DP, OR1D5, PMCHL1, CXCR4, TPM2, LIN28B, LOC440896, HFM1, PCSK9, OR5D16, POU1F1, TLR7, ROBO2, SPIRE2, PHGR1, CCDC39, DAOA-AS1, PROX1, OR1B1, LOC100506546, LINC00837, GABRA3, IRX6, DPPA3, LHX1, DNMT3B, S100A2, TRAF5, LOC100996254, DHX58, SPDYE1, PURG, LOC284837, GLT25D2, ARHGEF19, RPL22L1, LPAR6, DGKB, GYPA, DLK2, CNOT6, GAGE1, FAM154B, SMAP2, CLCA2, LRRN1, TAAR8, GRAP2, LCTL, SAA1, HIST1H2BG, TMEM144, LHFPL3, ANK1, CCR5, LOC100505903, LINC00410, PLXDC2, RAX2, GPR111, EN2, SRRM4, FGF7, METTL10, FAM129A, DCAF16, LOC389332, JAG1, INS, CFHR1, PARVA | DIP2C, FUS, PPP4C, AHSA1, RRP7A, MAD2L2, LSM4, CBWD1, DTD1, ARPC2, CAPNS1, MICA, MRPS2, TMEM177, YKT6, PSMB8, DNAJC9, NUP37, NUDCD2, BRK1, TGFB1, TFB1M, CDC37, GNB2, ASNA1, RTFDC1, TACC3, WDR82, COPE, SAP30, RNF141, PARL, BRIX1, SNRPB, C12orf10, AKR1A1, GSTK1, PPP2CB, HDLBP, BCAP31, LOC101060301, IGBP1, HSPA8, ZFAND2B, ID1, MCL1, FDPS, EIF3M, PLXNA1, AIP, HRAS, WIPF2, NUP153, PIH1D1, TEX10, EXOSC7, HN1L, NBEAL2, CLTA, IDH3B, ITPK1, EBPL, EIF4G1, IFT20, SLC32A1, LMAN2, POLR3H, OR10G2, C20orf27, GAPDH, PSMD10, ATXN7L3B, FXN, S100A16, FIP1L1, BANF1, ZFAND6, NAT14, MFAP1, PIGY, LRPAP1, VARS, IFRD2, RNF26, PLA2G4A, TIMMDC1, ILF2, CDK11B, TSPYL4, BRD3, PAXIP1, NMRAL1, TMEM14B, ARV1, TMEM223, OSTF1, TXNDC5, CDIPT, PEBP1, LOC732265, EFHD2, CENPA, CCDC101, APBB3, YTHDC1, HIP1, RBCK1, PSMB10, LOC401397, S100A16, EIF2C2, DHCR24, FUNDC2, MRPL32, MPDU1, MED27, FAT1, TOMM34, APOBEC3C, HNRNPH2, CNPY3, FIBP, ACN9, CAMKK2, SUCLG1, USP1, SMARCA5, LAP3, HAUS1, LOC101060625, UBIAD1, VPS13C, MTCH1, MRPL12, CCDC124, C1orf52, TAF1D, EIF3I, RPS19BP1, TOMM70A, ACOT8, NOP56, CTDP1, UBA1, UQCRC1, DHX33, FDPS, PARL, OARD1, C17orf75, C7orf49, PDCD6, PQBP1, PPP4R1, RBM14, NUDT19, CCDC24, EMC9, WDR20, TMEM214, TRIB3, RNASEH2A, DIP2A, ID3, LYPLA2, TRUB2, LOC101060198, CLN3, PABPC4, ZNF239, SUDS3, RTN3, ZBTB5, ZNF511, C6orf70, NSMCE4A, SSR2, HIST3H2A, CD99, UNC50, HMGN4, C4orf27, BUB3, ATP6V0C, FAM213B, UBN1, FHOD1, DTYMK, RHOB, PSMG3, CYB5D2, DNTTIP1, SLC25A24, FBXL5, HPCAL1, PAK4, VPS26A, SUMO1, LOC100288142, MED22, TMEM184B, EHBP1L1, RDH13, OR4D9, SLC25A39, KRTCAP3, C8orf33, ACN9, AURKB, MRPL2, TEAD4 |
