# Supplementary material for: In Vitro and In Silico Mechanistic Insights into miR-21-5p-Mediated Topoisomerase Drug Resistance in Human Colorectal Cancer Cells
Source: Biomolecules. 2019 Sep 9;9(9):467. doi: 10.3390/biom9090467 (PMC6769444; doi:10.3390/biom9090467)
Supplement: Supplementary file 1 [file biomolecules-09-00467-s001.zip › Table S2-proteasome genes.docx]

**Table S2. Gene list for KEGG proteasome pathway mapping.** Differentially expressed genes (DEGs) in DLD-1-miR-21 vs. DLD-1-vector cells were subject to a KEGG pathway enrichment analysis. Genes responsible for KEGG proteasome pathway mapping (hsa03050:Proteasome) are shown in this table.

| **KEGG ID** | **Gene symbol** | **Description** | **Log2 ratio** | ***p* value** |
| --- | --- | --- | --- | --- |
| PA200 | PSME4 | proteasome (prosome, macropain) activator subunit 4 | 1.68 | 0.02754201 |
| Rpn15 | SHFM1 | split hand/foot malformation (ectrodactyly) type 1 | 1.29 | 2.14117E-12 |
| βli | PSMB9 | proteasome (prosome, macropain) subunit, beta type, 9 (large multifunctional peptidase 2) | 1.18 | 1.97331E-05 |
| α1 | PSMA2 | proteasome (prosome, macropain) subunit, alpha type, 2 | -1.07 | 2.53044E-05 |
| Rpn7 | PSMD6 | proteasome (prosome, macropain) 26S subunit, non-ATPase, 6 | -1.09 | 3.32144E-06 |
| PA28γ | PSME3 | proteasome (prosome, macropain) activator subunit 3 (PA28 gamma; Ki) | -1.10 | 0.008711034 |
| β7 | PSMB4 | proteasome (prosome, macropain) subunit, beta type, 4 | -1.24 | 2.94788E-06 |
| β3 | PSMB3 | proteasome (prosome, macropain) subunit, beta type, 3 | -1.30 | 3.56883E-09 |
| Rpt6 | PSMC5 | proteasome (prosome, macropain) 26S subunit, ATPase, 5 | -1.43 | 3.40608E-15 |
| α7 | PSMA3 | proteasome (prosome, macropain) subunit, alpha type, 3 | -1.52 | 1.49863E-11 |
| Rpt4 | PSMC6 | proteasome (prosome, macropain) 26S subunit, ATPase, 6 | -1.60 | 3.16886E-08 |
| β5 | PSMB5 | proteasome (prosome, macropain) subunit, beta type, 5 | -1.60 | 5.85077E-17 |
| Rpt3 | PSMC4 | proteasome (prosome, macropain) 26S subunit, ATPase, 4 | -1.66 | 1.14981E-12 |
| α6 | PSMA1 | proteasome (prosome, macropain) subunit, alpha type, 1 | -1.75 | 1.9685E-17 |
| PA28α | PSME1 | proteasome (prosome, macropain) activator subunit 1 (PA28 alpha) | -1.87 | 8.03852E-11 |
| β2 | PSMB2 | proteasome (prosome, macropain) subunit, beta type, 2 | -2.29 | 8.95487E-18 |
| Rpn6 | PSMD11 | proteasome (prosome, macropain) 26S subunit, non-ATPase, 11 | -2.39 | 4.14533E-15 |
| PI31 | PSMF1 | proteasome (prosome, macropain) inhibitor subunit 1 (PI31) | -2.50 | 9.16105E-14 |
| Rpn3 | PSMD3 | proteasome (prosome, macropain) 26S subunit, non-ATPase, 3 | -2.57 | 7.66822E-07 |
| Rpn10 | PSMD4 | proteasome (prosome, macropain) 26S subunit, non-ATPase, 4 | -2.85 | 4.44289E-10 |
| Rpt5 | PSMC3 | proteasome (prosome, macropain) 26S subunit, ATPase, 3 | -2.87 | 8.93555E-14 |
| Rpt1 | PSMC2 | proteasome (prosome, macropain) 26S subunit, ATPase, 2 | -2.94 | 1.53571E-13 |
| β2i | PSMB10 | proteasome (prosome, macropain) subunit, beta type, 10 | -3.40 | 1.01338E-12 |
| β5i | PSMB8 | proteasome (prosome, macropain) subunit, beta type, 8 (large multifunctional peptidase 7) | -4.407 | 2.01441E-28 |
