# Supplementary figures and images for: In Vitro and In Silico Mechanistic Insights into miR-21-5p-Mediated Topoisomerase Drug Resistance in Human Colorectal Cancer Cells
Source: Biomolecules. 2019 Sep 9;9(9):467. doi: 10.3390/biom9090467 (PMC6769444; doi:10.3390/biom9090467)

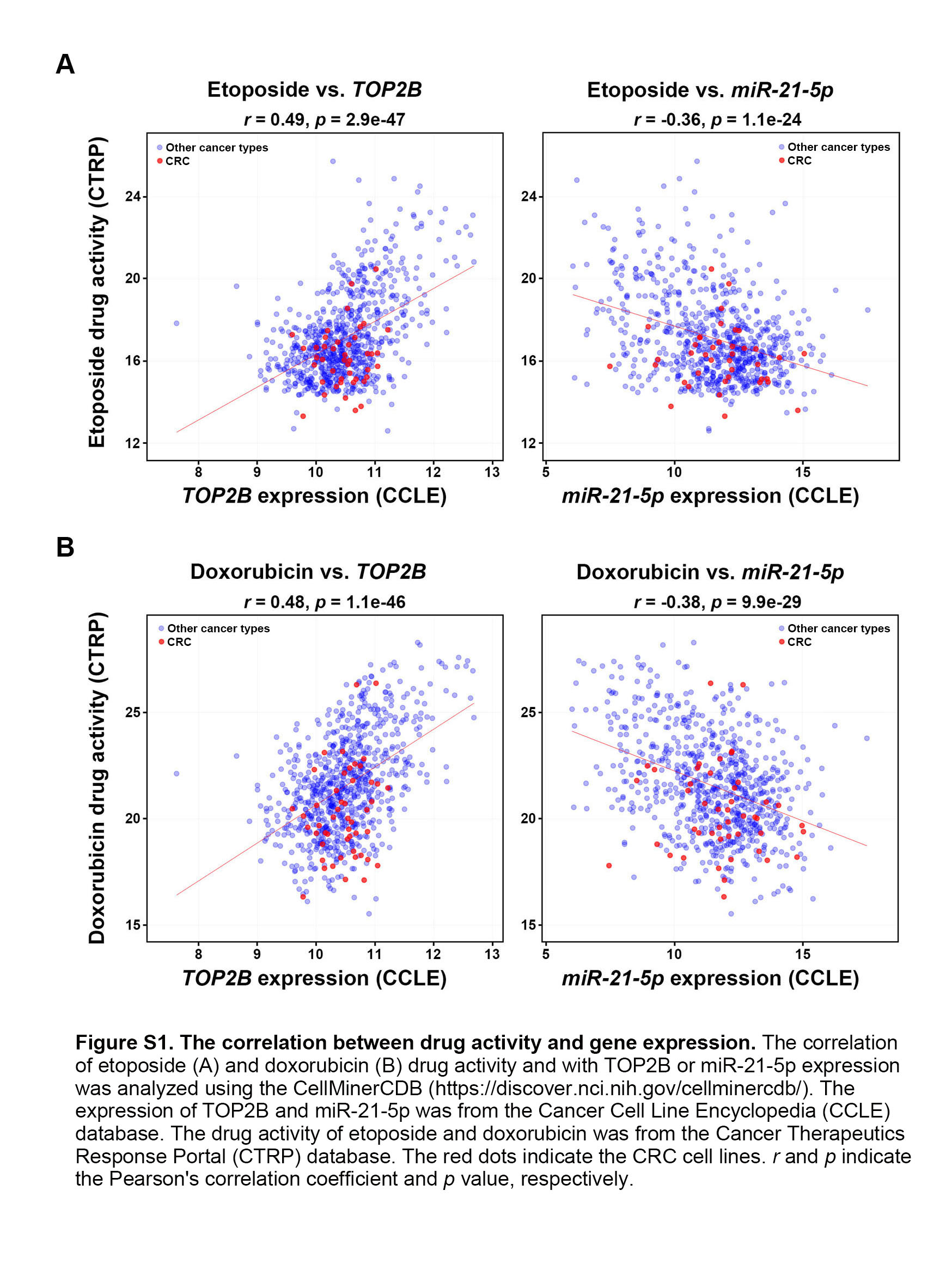

Supplement: Supplementary file 1 [file biomolecules-09-00467-s001.zip › FigS1.jpg]

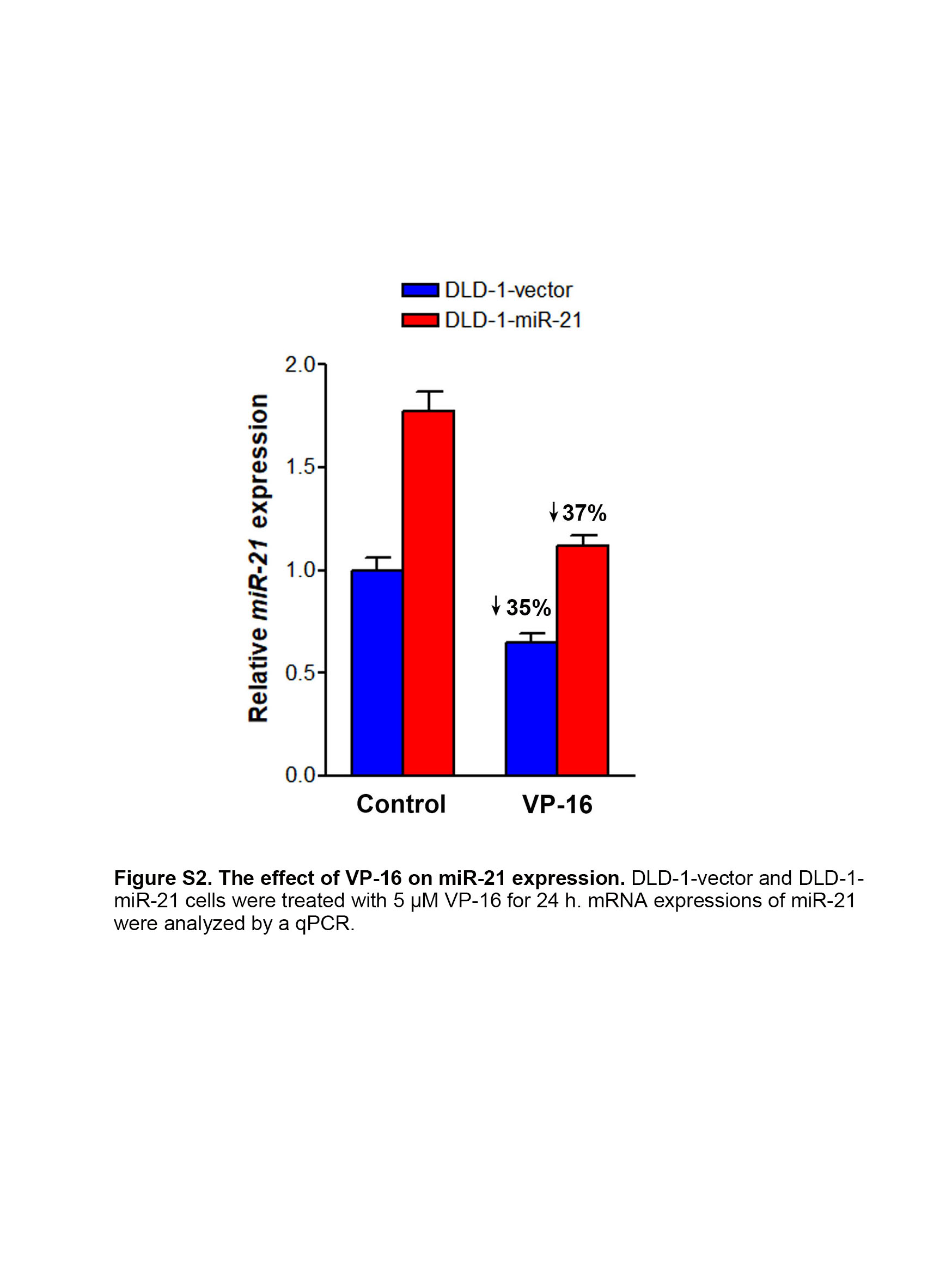

Supplement: Supplementary file 1 [file biomolecules-09-00467-s001.zip › FigS2.jpg]

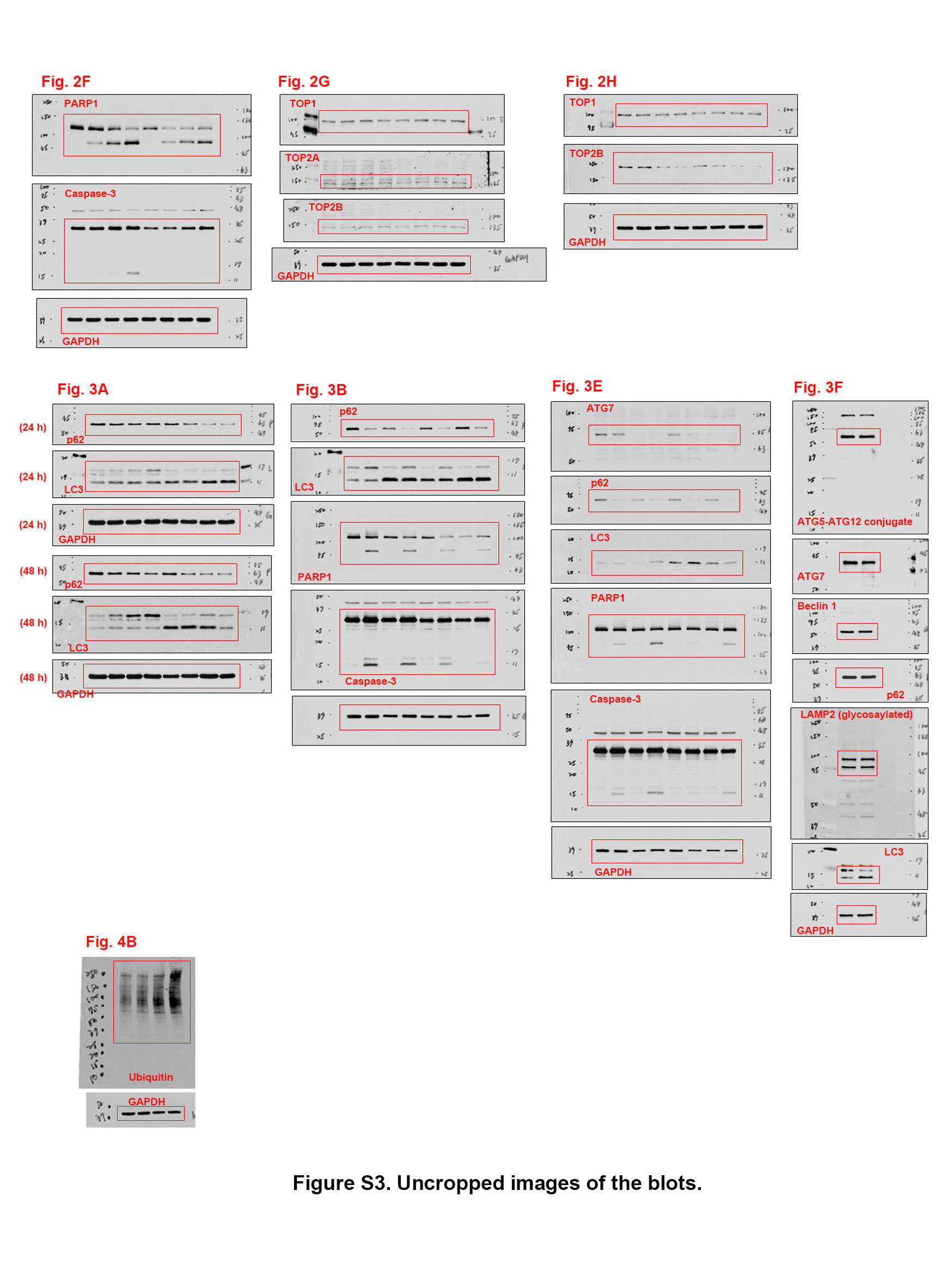

Supplement: Supplementary file 1 [file biomolecules-09-00467-s001.zip › FigS3.jpg]
